# Can we achieve the perfect injectable scaffold for cell therapy?

**DOI:** 10.4155/fsoa-2017-0153

**Published:** 2018-01-25

**Authors:** Capucine Guyot, Sophie Lerouge

**Affiliations:** 1 Department of Mechanical Engineering, École de Technologie Supérieure (ETS), 1100 Notre-Dame West, Montréal, QC H3C 1K3, Canada; 2 Laboratory of Endovascular Implants & Biomaterials (LBEV), Centre de Recherche du CHUM (CRCHUM), 900 St Denis, Montréal, QC H2X 0A9, Canada

**Keywords:** biomaterials, cell therapy, design, drug delivery, injectable scaffolds, tissue engineering

Following the tremendous development of injectable scaffolds for drug delivery, as well as recent breakthroughs in both cell therapy and tissue engineering, there is now a growing need for injectable scaffolds designed to host cells, safely deliver them without losses and protect them *in situ*.

## Why?

It is a well-known fact that when cells are injected straight into tissues, their therapeutic effect is strongly limited by their poor retention and dramatically low survival rate [1]. Using biomaterials as scaffolds for cell encapsulation significantly helps overcoming those two critical issues.

## What for?

Once safely delivered at the targeted location, encapsulated cells can either be used for cell therapy or *in situ* tissue engineering. They may either induce the desired therapeutic effect by paracrine delivery of various biomolecules (growth factors, cytokines, hormones, etc.), like in heart regeneration with encapsulated stem cells [2], or replace the damaged cells and stay in place long enough to regenerate the tissue. In the latter case, the scaffold acts as a physical support for cell growth and extracellular matrix (ECM) deposition until complete degradation of the material. This is advantageous when renewing tissues with specific mechanical properties such as cartilage, bone [3,4] and intervertebral disc, etc. In a few cases, cells are expected to leave the matrix and reach surrounding tissues. This is the case for instance of the T-lymphocyte-delivering scaffolds developed by our team for cancer immunotherapy, where immune cells can escape and attack surrounding tumors [5].

## Why injectable scaffolds?

Injectable technologies show significant advantages over preformed scaffolds. First of all, they allow for minimally invasive delivery through needles and catheters, which do not require open surgery, thus reducing surgery-associated risks as well as convalescence time. Then, since they flow, they can properly fill irregular defects where preformed materials cannot adjust to the tissue topology. Being able to go deeper with endoscopes or catheters also facilitates access to frail tissues and critical regions with associated morbidity. Finally, they could also possibly reduce treatment's duration and its related costs by minimizing *in vitro* preparation steps. When cells are cultured outside the body, they have to be supplied with bioactive factors, whereas when injected they can shortly benefit from the environmental cues, such as the bioactive factors released by the surrounding living organisms.

## Micro- versus macroencapsulation

Several different technologies enable injection of cells within a biomaterial. Microencapsulation, which consists of encapsulating cells in microspheres prior to injection, is increasingly used to achieve high cell retention or protect the cells from the immune system (for example, when working with pancreatic cells for diabetic patients [6]). However, we will focus in this editorial on macroencapsulation. Cells are loaded in a macroscale amount of material, liquid upon injection, which is expected to solidify *in situ* later on while entrapping the cells.

## How?

In addition to conventional biocompatibility issues, such as ensuring the absence of toxic, immunogenic and mutagenic products and by-products, many specific requisites have to be taken into account in order to create an ideal injectable cell-containing scaffold. Those are also called design criteria and are listed below.

- Injectability. The material should be in a sufficiently fluidic state to be administrated through a needle or a catheter, which diameter depends on the targeted tissue; hence, it should either be a low-viscosity solution or exhibit a shear-thinning behavior upon injection.
- Sustainable mechanical properties. Once injected, the material should rapidly get cohesive to avoid dispersion and migration. In that sense, gelation or polymerization should significantly start or even be completed shortly after injection. Mechanical properties should be sufficient to withstand biological forces [7], which is noticeably challenging for tissues undergoing dynamic loading (such as cartilage, bone, etc.). Stimuli-responsive materials like temperature and pH-sensitive polymers are particularly interesting in that sense [8].
- Significant shrinkage should also not take place during 3D-network formation, in order to perfectly fill the defect.
- Compatibility with cell encapsulation and/or entrapment. The scaffold should provide a friendly environment for the cells to survive, grow and achieve their desired function.
  - Cell encapsulation should be homogeneous, easy and gentle. The scaffold has to protect the cells from shear forces inherent to injection, which can be a potent cause of mortality. When using hydrogels, their rheological properties strongly influence how cells spread and survive shear stresses [9].
  - The material should provide an environment close to the cell's physiological conditions, namely high water content, neutral pH (7.0–7.4) and threshold-limited osmolality (generally around 300 mOs/ml, though this value can slightly vary among tissues).
  - The scaffold should never hold any kind of toxic compound, which could be detrimental to both encapsulated cells and surrounding tissue. Hence the least possible of chemical initiators, crosslinkers or radiation during polymerization should be used. The biomaterial should also induce few inflammations, avoiding formation of a fibrous tissue around the implant that would impair cell and nutrients transfer.
  - Nutrients, oxygen and cell waste products should be able to transfer through the matrix. Transfer efficiency is dictated by the scaffold's porosity, and possibly backed by diffusion-promoting mechanisms (water movement, etc.). However, neovascularization of the scaffold is mandatory when aiming for long-term viability of the cells, except for some rare cases. It is consensus that a cell has to be <200 μm away from a vessel in order to ensure ideal nutrient access and excellent survival rate [10].
  - Both cell–cell and cell–matrix interactions have a significant impact on cell morphology, viability and function, but from one cell source to another, one interaction type may be predominant. For anchorage-dependent cells, cell–matrix interactions must be promoted by the material itself, either by choosing a material that inherently enhances them, like collagen, or by chemical modification. As an example, chemical grafting of cell-binding groups can be achieved with arginylglycylaspartic acid (RGD)-peptides, which are recognized by integrins [11]. Cell–matrix interactions also influence how cells proliferate and, if applicable, differentiate. Adding bioactive compounds such as growth factors or drugs within the scaffold is another mean to boost the desired cell function or differentiation [12]. In some cases, cell–cell interactions are crucial for cell survival and function. Scaffolds’ porosity should then be sufficient to allow for 3D colonies formation.
  - Depending on the targeted application, cells should either be able to escape the matrix after a given amount of time in order to reach surrounding tissues, or be kept inside the implant, where they will generate new ECM and grow into neotissue.
- Biodegradability. Whether the scaffold is meant for cell therapy or tissue engineering, it must be biodegradable in order to be eliminated once not needed any longer. In the case of tissue regeneration, the degradation rate should match cells ECM production. Byproducts of the degradation should be nontoxic and eliminated by biological pathways.
- Tissue adhesion. Tissue adhesive properties can help increasing scaffold retention on targeted tissues, and possibly ease cell transfer and paracrine factors circulation between the host tissue and the matrix. There are many ways to increase a biomaterial's tissue adhesiveness. Chemical grafting of highly adhesive compounds makes adhesion more specific and especially efficient. For instance, thiol grafting has increased several biopolymers’ adhesion from two- to 140-times [13]. Catechol grafting, inspired by marine mussels DOPA-mediated adhesion, is another powerful way to drastically enhance wet surfaces adhesiveness [14].

## When?

Meeting all the design criteria with a single biomaterial is still an issue as of today. Hydrogels, which can be made either from natural or synthetic polymers, appear to be candidates of choice due to their high water content as well as structural similarities to the ECM. Many of them are formed under mild conditions, which provide the adequate environment for cytocompatible cell encapsulation. However, their mechanical properties are generally poor, especially when avoiding chemical crosslinking methods.

Yet, in the past 10 years, significant improvement has arisen on that matter. Injectable, cell-encapsulating interpenetrating polymer networks, composed of two polymer networks that can independently and simultaneously crosslink to form hydrogels in a cell-friendly manner, have been developed [15,16]. Thermosensitive hydrogels, which undergo a sol-gel transition upon heating to body temperature, can now achieve high mechanical properties *in situ* thanks to the use of new gelling agents combinations [17]. Self-healing hydrogels, crosslinked by unsteady bonds which form and break continuously, are also promising injectable scaffolds. This unique property allows them to be squeezed in a catheter's narrow diameter and easily injected, before reshaping once on the targeted site. Some are now compatible with cell encapsulation [18]. Combining recent progress in material science, knowledge in cell biology and the tremendous opportunities offered by stem cells and induced pluripotent stem cells should significantly improve the outcomes of cell therapy in the next decade. Additionally, these injectable scaffolds could also be used as bioinks for 3D bioprinting, an emerging and rapidly growing field in building cell-hosting tridimensional structures [19]. However, care must also be taken to design products that would eventually be approved by regulatory laws, and practically usable by clinicians.
